# Unique Findings and Novel Treatment Strategy of a Giant Coronary Artery Aneurysm Associated with a Significant Pericardial Effusion

**DOI:** 10.1155/2020/8890806

**Published:** 2020-06-09

**Authors:** Osayi Lawani, Rick Ganim

**Affiliations:** ^1^HCA Houston Healthcare, Kingwood, USA; ^2^Vital Heart & Vein, HCA Houston Healthcare, Kingwood, USA

## Abstract

Giant coronary artery aneurysms are a rare, asymptomatic occurrence. Presently, there is a lack of substantial research performed in the U.S., likely due to its low prevalence. As we are increasingly becoming a global community, strengthening data for seemingly rare disease processes such as this need to be addressed, particularly when they can progress to involve complications such as pericardial effusion caused by aneurysmal rupture or infection. A popular treatment option for these aneurysms is polytetrafluoroethylene-covered stents; they have been favorable with obtaining a high percentage of procedural success rates in aneurysms not associated with myocardial infarctions. In this paper, we present a case of a giant coronary artery aneurysm located in the left circumflex coronary artery that was complicated by a pericardial effusion. We will also present its unusual repair using a long drug-eluting stent as a scaffold to overlap covered coronary stents used to help exclude the aneurysm.

## 1. Introduction

**Figure 1:**
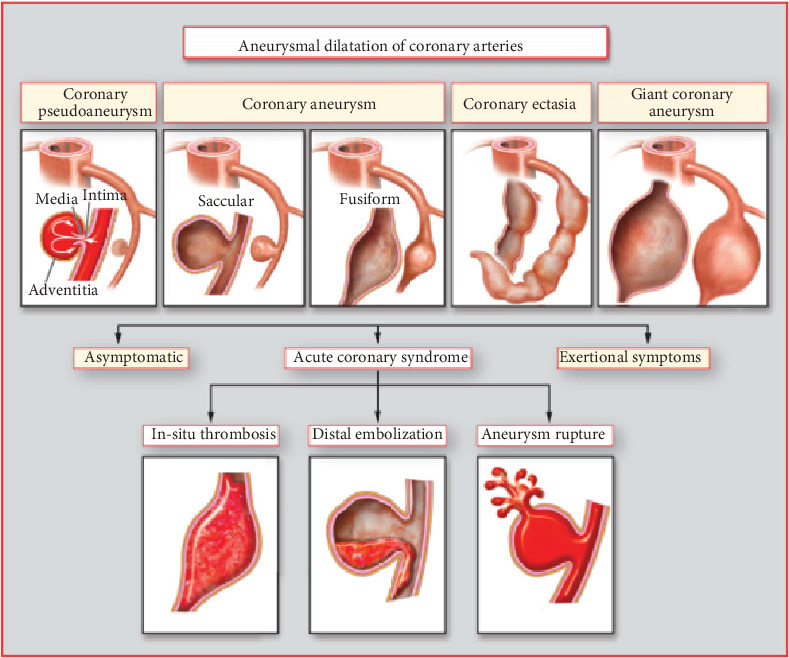
Morphologies of coronary artery aneurysms [2].

Coronary artery aneurysms (CAAs) or ectasias are an unusual cardiac finding. As it is seen more often in males, the incidence for patients undergoing diagnostic imaging with incidental findings is currently between 0.3 to 5.3% [1]. Occurrence may increase due to the widespread use of computed tomography angiography in clinical practice. CAAs are described as a 1.5-fold dilation in a localized segment of a coronary artery in correlation to adjacent segments of the same vessel, although dilations can also be diffused [1]. Additionally, its morphology can be classified as either saccular, fusiform, or mixed (Figure 1) [2].

Italian physician Giovanni Battista Morgagni first described a giant CAA in 1761, where he relays the discovery of “an aneurysm the size of a walnut that had ruptured into the pericardium,” during a postmortem evaluation [3]. CAAs are classified as “giant” when their diameter is greater than 8 mm or 4 times the reference vessel diameter [4]. Options for treatment consist of either percutaneous intervention, surgical excision, coronary artery bypass grafting, or conservative medical management. The aim of this paper is to describe challenges associated with providing intervention for giant CAAs. In this report, we present a case of a middle-aged male with multiple CAAs, which was also complicated with the development of a giant aneurysm and an associated pericardial effusion.

## 2. Case History

A 56-year-old male with a past medical history of coronary artery disease with stents, hypertension, hyperlipidemia, end-stage renal disease on hemodialysis, renal transplant, and prostate cancer with radiation presented with substernal chest pain and shortness of breath during an outpatient appointment.

Seven years earlier, the patient presented to the emergency department with similar symptoms. Electrocardiogram showed normal sinus rhythm with ST abnormalities, and he was diagnosed with unstable angina. An echocardiogram showed an ejection fraction of 45-50%, left ventricular hypertrophy, and left ventricle septal apical wall hypokinesis. Nuclear perfusion testing revealed moderate anteroseptal ischemia and decreased left ventricular systolic function, with an ejection fraction of 40%. A left heart catheterization was performed and showed diffuse ectasia of all three major coronary arteries. The left main had mild plaquing without any significant stenosis. The left anterior descending was large-caliber, and the mid-segment had eccentric 95-99% stenosis. The mid-to-distal left anterior descending had diffuse ectasia, as well as approximately 40% stenosis in its distal segment. The left circumflex artery was diffusely ectatic throughout. The right coronary artery was moderate in caliber, and the proximal segment and the posterolateral branch were diffusely ectatic without any critical stenosis. A percutaneous transluminal coronary angioplasty was performed with stent placement in the mid-left anterior descending with a 4.0 x 15 mm bare-metal integrity stent. Electrocardiogram following the procedure showed a normal sinus rhythm. The patient was discharged home on dual antiplatelet therapy and aggressive blood pressure and lipid control therapy.

**Figure 2:**
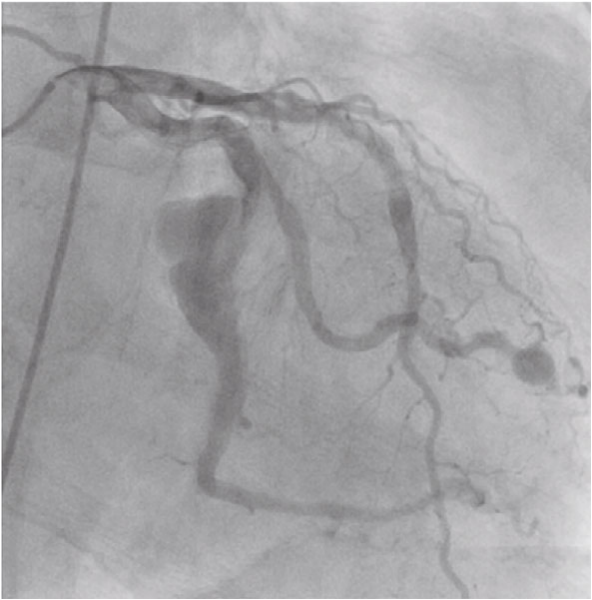
Giant coronary artery aneurysm in left circumflex coronary artery.

Five years later, the patient presented to the emergency department after experiencing progressive shortness of breath, chest pressure, and lightheadedness for one week. Due to his history of unstable angina, a left heart catheterization was performed, and it was found that the left main coronary artery had no critical stenosis. Stent patency persisted in the mid-left anterior descending, and 40% stenosis in its mid-segment was consistent with the previous left heart catheterization. Within the left circumflex artery, the first obtuse marginal branch had a small aneurysm in its distal segment, and distal to the first take off, a very large CAA with sluggish flow was appreciated (Figure 2). An echocardiogram also revealed a large pericardial effusion with early tamponade physiology. Cardiovascular surgery was consulted, and the patient underwent urgent removal via a pericardial window. 1,100 ml of pericardial fluid was removed and was surmised to have originated from a rupture of the giant CAA.

**Figure 3:**
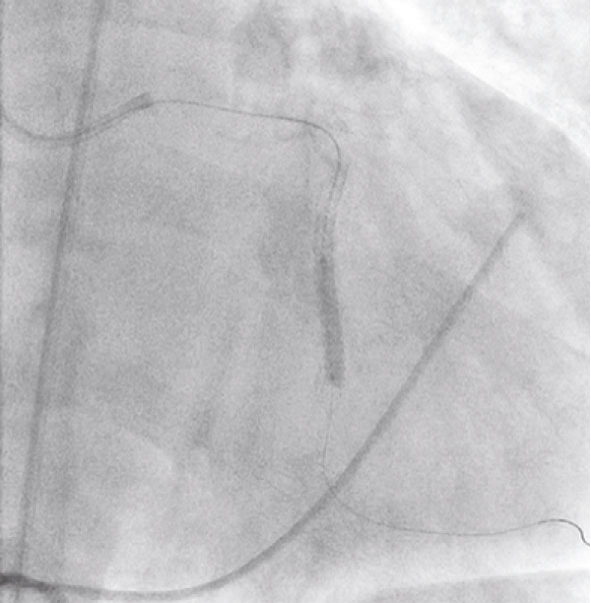
Synergy stent placement proximal and distal to a giant coronary artery aneurysm for support.

The following day the patient underwent a left heart catheterization of the left circumflex artery with plans to place a JOSTENT GraftMaster stent after receiving IRB and Abbott Vascular approval, due to its off-label use. The patient also consented to undergo covered stent placement for the aneurysm. A 7-french sheath was placed in the left femoral artery. A 7-french XB 3.5 guide catheter was used to engage the left main coronary artery, and a Luge wire was advanced into the distal obtuse marginal branch. Following intravenous anticoagulation, intravenous ultrasound was then advanced over the Luge wire and utilized to identify the size and length of the segment of the artery proximal and distal to the large aneurysm. Unexpectedly, the length of the aneurysm was longer than the longest GraftMaster covered stent (26 mm); intravenous ultrasound measured the length of the aneurysm at 32 mm. A 3.0 x 38 mm Synergy stent was then positioned proximal and distal to the aneurysm to provide support for placement of the covered stents. Next, the stent was postdilated with a 3.5 x 12 mm NC Emerge balloon and a 4.0 x 26 mm GraftMaster covered stent was positioned in the mid-to-distal segment of the aneurysm and was deployed at 15 atmospheres. A 2.8 x 16 mm GraftMaster covered stent was positioned in the proximal segment of the aneurysm and just proximal to the segment to the native coronary artery and was deployed at 16 atmospheres (Figure 3). The mid-to-distal segment was postdilated with a 4.0 x 20 mm NC Emerge balloon up to 16 atmospheres. Finally, the very proximal segment of the stent was postdilated with a 3.0 x 12 mm NC Emerge balloon.

**Figure 4:**
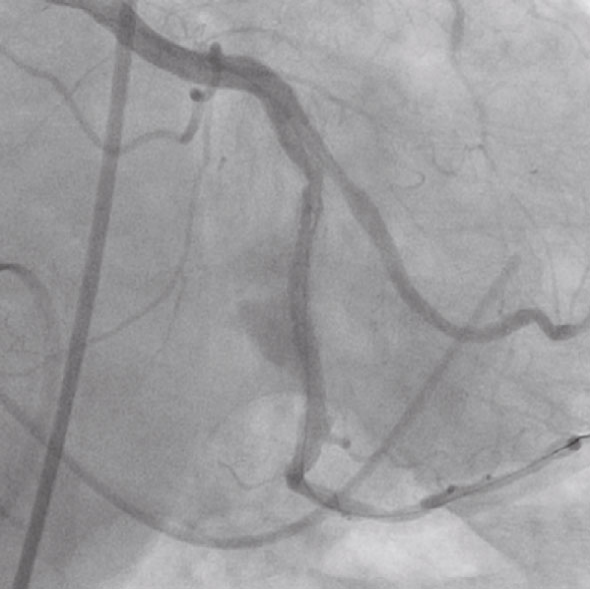
Resolution of a giant coronary artery aneurysm following repair with GraftMaster covered stents.

Postintervention angiography revealed a resolution and complete sealing of the giant aneurysm with a Thrombolysis in Myocardial Infarction blood flow score of 3 (Figure 4). An echocardiogram showed an ejection fraction of 55-59% with no evidence of pericardial effusion. Electrocardiogram postprocedure displayed normal sinus rhythm with left axis deviation and left ventricular hypertrophy. The patient recovered well, and he was discharged a few days later on dual antiplatelet therapy.

**Figure 5:**
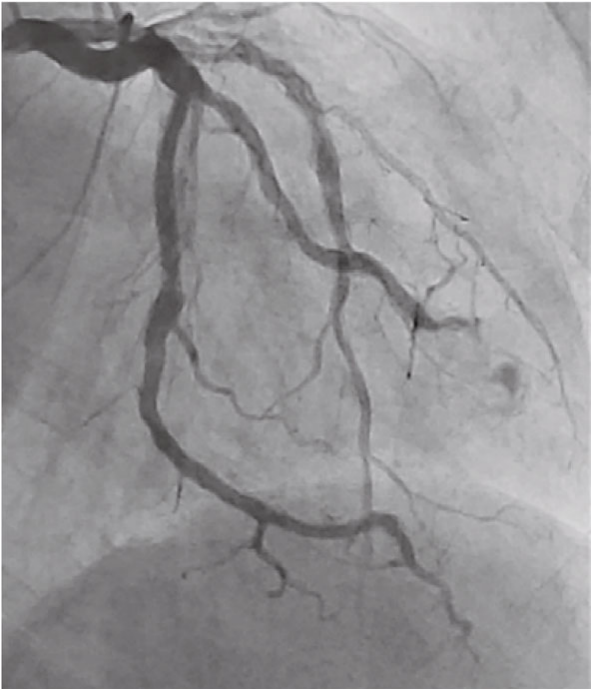
Continued resolution of a giant coronary artery aneurysm two years later.

Two years later, the patient presented with complaints of chest pain and shortness of breath during an outpatient visit. Following an abnormal stress test, a left heart catheterization was performed. It was found that the previously treated left circumflex artery stent was widely patent with continued resolution of the giant aneurysm (Figure 5). Previous stents in the midsegment of the left anterior descending also remained patent. New findings included 95% stenosis and ectasia of the mid posterolateral branch of the right coronary artery, and 75-80% stenosis in the left circumflex artery distal to the repaired aneurysmal lesion. Percutaneous intervention was performed, and a 3.0 x 16 mm Synergy stent was placed distal to the previously treated aneurysm. Also, a 3.5 x 16 mm Synergy stent was placed in the mid posterolateral branch. Following the procedure, he was discharged home to continue long-term dual antiplatelet therapy.

## 3. Discussion

Since Dr. Charles Bougon published the first clinical description of CAAs in 1812 following a postmortem discovery, findings from autopsies are the basis for most of the knowledge on CAAs due to often treating them with percutaneous intervention or by minimal tissue excision [5]. CAAs are usually found in the right coronary artery, followed by the left anterior descending, left circumflex artery, and the left main. The most common cause of CAA is atherosclerosis in North America and Europe, and Kawasaki disease in Japan and China. Other causes include inflammatory disorders, trauma, bacterial endocarditis, and complications following percutaneous intervention. Most of all patients with the atherosclerosis type will present with greater than 75% narrowing of the artery [6]. Some presentations will have multiple small aneurysms. The development of atherosclerotic CAA will usually mirror clinical features and risk factors related to coronary artery disease. Symptoms of angina will correlate with areas related to coronary artery stenosis, and consequently, aneurysm.

Coronary computed tomography angiography is currently the best modality for imaging of atherosclerotic CAA due to being able to perform a complete evaluation of coronary artery plaques, vessel patency, and overall visualization of the actual aneurysm and any resulting thrombus formation [7]. During cardiac catheterization, aneurysms will likely be underestimated because there is only an endoluminal view [7]. Although definitive diagnosis requires a histopathological sample, it is difficult to obtain unless the patient is postmortem or open-heart surgery is performed.

Presently, data is lacking for the prevalence of giant CAAs in the United States. There has been a slight increase in research; however, expanding data is primarily in Asiatic countries likely due to having a higher incidence of Kawasaki disease, which is endemic in this population [8]. Furthermore, prevalence data for giant CAAs is practically nonexistent to be able to predict the possible incidence in the United States population, independent of knowledge of known risk factors. Between 2005 to 2011, there were only a few angiography studies in the United States which reported a 0.2% CAA prevalence, whereas in Taiwan, it was found that there were approximately 200 cases per year within that time frame, with no increase in the incidence of CAA [8]. Another report speculated that the occurrence of CAA may be due to a complication of angioplasty and drug-eluting stent implantation [6]. As one would gather, treatment of this condition, particularly if it is giant, is currently done on a case-by-case basis.

Rupture of an aneurysmal coronary artery can lead to thrombus formation, embolization, ischemia, acute pericarditis, or pericardial effusion, which may progress to cardiac tamponade, as was seen in this case [9]. Incidence and prognosis of these phenomena are currently unknown. What has been noted is that the 5-year survival rate was 71% for aneurysms greater than 8 mm in diameter, and that the prognosis of CAAs greater than 20 mm is currently unknown and are at an increased risk of rupture [10]. Other complications noted were mycotic aneurysms that followed a myocardial ischemia and increased incidence in the septic or the immunecompromised [10].

**Figure 6:**
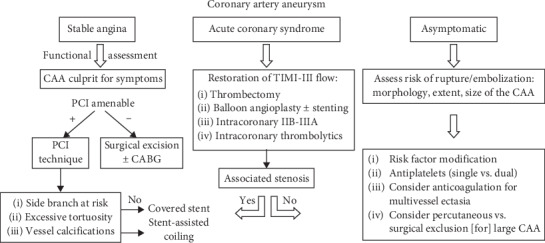
Suggested algorithm for management of coronary artery aneurysms [2].

Assessment and treatment for CAA is not currently standardized; however, asymptomatic patients with aneurysms should receive treatment, especially if they are greater than 30 mm in diameter [1]. Appropriate therapy is uncertain. Current options are surgical resection, coil embolization, and percutaneous approaches with covered stents, and pharmacotherapy (Figure 6) [2]. There is a question as to whether conservative management or surgery is the best approach. Two common interventions are coronary artery bypass grafting that may or may not include aneurysm ligation or resection and polytetrafluoroethylene-covered stents [11]. One study reported that polytetrafluoroethylene-covered stents are effective for aneurysms of 6-10 mm in diameter. Surgical intervention is the first line when the left main is involved or if the aneurysm is “giant” [2]. In this case, a JOSTENT GraftMaster coronary stent graft system was used, as surgical repair was not found to be a viable option. It consists of a single polytetrafluoroethylene layer that is sandwiched between two coaxial, 316 L stainless steel, slotted-tube, balloon-expandable stents [12]. It is available for diameters of 2.75-5 mm, lengths of 12, 16, 19, and 26 mm, and has an overall 96% procedure success rate when it is purposed for its intended manufactured use [12]. Strategic management of CAA is not definitive and should be coordinated based on each individual patient and their clinical findings until further research data is available to provide a cohesive approach to therapy.

## 4. Conclusion

The diagnosis and management of giant CAAs are challenging. Detailed physical examination and careful interpretation of chest X-ray and/or computed tomography angiography findings are extremely important in asymptomatic patients or in those who have complaints of chest pain. Treatment with a percutaneous intervention-covered stent is the most common treatment method at this time, particularly since the outcomes after percutaneous intervention have been historically favorable when not associated with myocardial infarction. We described a unique case in how to intervene on a giant CAA that was unexpectedly longer than the longest covered stent in the setting of a pericardial effusion. Further research in the United States is essential to increase the knowledge on how CAAs are managed, with a standardized assessment that would lead to an early diagnosis of this rare disease process.

## Data Availability

Data is available upon request to the corresponding author.
